# Dynamics of *Salmonella* infection of macrophages at the single cell level

**DOI:** 10.1098/rsif.2012.0163

**Published:** 2012-05-02

**Authors:** Julia R. Gog, Alicia Murcia, Natan Osterman, Olivier Restif, Trevelyan J. McKinley, Mark Sheppard, Sarra Achouri, Bin Wei, Pietro Mastroeni, James L. N. Wood, Duncan J. Maskell, Pietro Cicuta, Clare E. Bryant

**Affiliations:** 1 Department of Applied Mathematics and Theoretical Physics, University of Cambridge, Cambridge CB3 0WA, UK; 2 Department of Veterinary Medicine, University of Cambridge, Cambridge CB3 0ES, UK; 3 Cavendish Laboratory, University of Cambridge, Cambridge CB3 0HE, UK

**Keywords:** *Salmonella*, macrophage, dynamic, infection rate, Holling's type II

## Abstract

*Salmonella enterica* causes a range of diseases. Salmonellae are intracellular parasites of macrophages, and the control of bacteria within these cells is critical to surviving an infection. The dynamics of the bacteria invading, surviving, proliferating in and killing macrophages are central to disease pathogenesis. Fundamentally important parameters, however, such as the cellular infection rate, have not previously been calculated. We used two independent approaches to calculate the macrophage infection rate: mathematical modelling of *Salmonella* infection experiments, and analysis of real-time video microscopy of infection events. Cells repeatedly encounter salmonellae, with the bacteria often remain associated with the macrophage for more than ten seconds. Once *Salmonella* encounters a macrophage, the probability of that bacterium infecting the cell is remarkably low: less than 5%. The macrophage population is heterogeneous in terms of its susceptibility to the first infection event. Once infected, a macrophage can undergo further infection events, but these reinfection events occur at a lower rate than that of the primary infection.

## 1. Introduction

The study of how cells are infected by bacteria forms the basis of cellular microbiology, and such studies have generated a wealth of knowledge about pathogenesis. *Salmonella enterica* subspecies *enterica* serovar Typhimurium (*Salmonella* Typhimurium) infects and survives within macrophages. To do this, *Salmonella* must adhere to, invade, survive within and proliferate within the cells, ultimately resulting in the death of host cells in many cases. Assaying the dynamic interactions between *Salmonella* and macrophages *in vivo* is technically challenging, and research relies on studying *S.* Typhimurium infections of macrophage populations *in vitro* [1]. These studies rely on gross measures of their outputs at the population level, such as changes in total bacterial number over time, the percentage of macrophage cells in a culture that die and the inflammatory responses induced in the macrophage population after infection [2–4]. Fine structure measurements of the individual steps in the *in vitro* infection system are lacking in the literature, and there are many assumptions made when analysing macrophage–*Salmonella* interactions. The assumptions include that the macrophage is highly susceptible to infection, and that all macrophages in a culture may become infected. While these assumptions appear plausible in many cases, it is by no means guaranteed that this is what is really happening in these systems.

The mechanisms involved in cellular infections by *S.* Typhimurium are complex. In epithelial cells, the invasion process is well understood and involves bacterial secretory system proteins, encoded by *Salmonella* pathogenicity island-1 (SPI-1) [5]. On the other hand, invasion of macrophages is primarily driven by phagocytosis, although SPI-1 proteins may also contribute [6]. After invasion, *S.* Typhimurium may be killed by the cell [7] or may proliferate within the *Salmonella*-containing vacuole (SCV), through the activity of proteins encoded by genes found in *Salmonella* pathogenicity island-2 [8]. Infection of macrophages by *S.* Typhimurium induces the production of pro-inflammatory mediators [4] and also leads to macrophage cell death [9]. These studies have been conducted mainly on populations of macrophages that are assumed to be able to readily infected, and therefore, the responses measured are all presumed to be from infected cells.

Here, we show that *S.* Typhimurium infection of macrophages occurs infrequently. Using quantitative analysis, we calculate for the first time, by two independent methods, that the probability of infection occurring after an initial contact between bacteria and macrophages is low. Infected cells can, however, undergo further infection events. Using a tight iterative coupling of experiment and theory, we show that the macrophage population is heterogeneous in terms of its susceptibility to the first infection event and that infection itself alters the rate of subsequent infections. We conclude that there are typically multiple contact events between salmonellae and macrophages before a cell becomes infected. Focussed studies on infection events in individual macrophages, rather than a simple analysis of the cell population response to infection, will lead to a reconsideration of mechanisms of pathogenesis and host resistance. This approach will be important for future development of novel intervention strategies for invasive salmonellosis and other intracellular pathogens.

## 2. Results

### 2.1. Preliminary models and testing basic assumptions

*Salmonella* infects macrophages, but how frequently this occurs or the probability that cells become infected is unknown. To calculate the infection rate of macrophages by *S.* Typhimurium, we first developed simple compartmental models representing macrophages (both infected and uninfected) and bacteria (both intracellular and extracellular) to define the basic dynamic events. These early rounds of models were extremely simplistic but served to underline ambiguities in existing knowledge. In the iterative process between models and experiments, we raised and tested a number of candidate assumptions, which were necessary for making parsimonious models. A basic assumption is that all macrophages can be infected when challenged with *S.* Typhimurium. To test this, murine-cultured primary-bone-marrow-derived macrophages (BMDMs) were infected with *S.* Typhimurium SL1344 constitutively expressing green fluorescent protein (GFP), referred to subsequently as G, at a multiplicity of infection (MOI) of 10. The bacteria were grown to late log phase to ensure the expression of SPI-1 genes, so that both the invasive and the phagocytic mechanisms of infection could occur. The lipopolysaccharide (LPS) O-antigen of extracellular bacteria was immunolocalized so as to discriminate between intracellular and extracellular *S.* Typhimurium. At 10 min post challenge (p.i.) with bacteria, 339 of 1500 BMDM were infected. As *S.* Typhimurium is an intracellular parasite of macrophages, we expected that most, and possibly all of the macrophages would be infected, which was not the case. To determine whether some cells are entirely resistant to infection, we performed experiments using increasing MOIs, and found that only at extreme MOIs is it possible to observe nearly all of the cells becoming infected. For example, 1483 of 1500 cells become infected by 10 min p.i. using an MOI of 800.

We explored the basic cellular dynamics, using a combination of results in the existing literature and the experiments in our system. We assumed that no macrophage proliferation occurs during the time course of a 3 h experiment, which is reasonable given that one round of BMDM cell division takes at least 24 h and is slowed in the presence of bacteria [10]. This assumption was supported by our cell viability counts during a 3 h period, which showed that there was no macrophage proliferation. Cell death is a well-known consequence of macrophage infection by *S.* Typhimurium [3] and presumably facilitates bacterial dispersion. In our experiments, minimal levels of cell death were detected up to 30 min after infection.

Formation of the preliminary model required a number of assumptions to be made, but also raised a number of important questions about what events occur during cellular infection that need to be answered in order to formulate an accurate mathematical model. A key question that arose was whether an infected macrophage could undergo further infection events. After the initial infection of a macrophage, the number of bacteria within the macrophage increases over time, and this is thought to be exclusively due to intracellular bacterial growth [8,11]. However, repeated infection of the same macrophage could also contribute to an observed increase in intracellular bacterial numbers. The question of whether cells, once infected, become refractory to further infection, or whether they might be reinfected has not been much considered, yet could have a significant impact on the number of intracellular bacteria.

**Figure 1.**
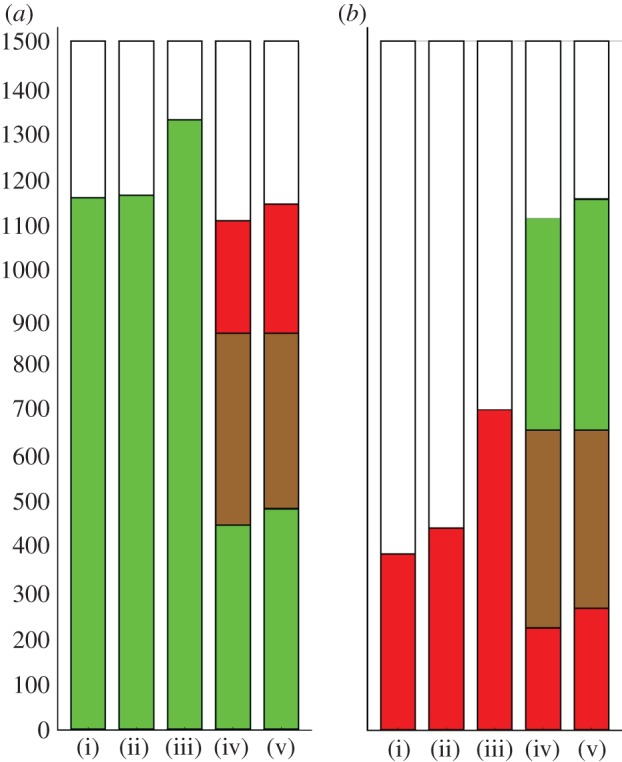
Sequential challenge of macrophages with G and R bacteria. Each bar represents 1500 BMDM, pooled from three separate replicates of 500 BMDM, and the colours show the number of BMDM infected by G (green), R (red), both G and R (brown) or uninfected (white). Each challenge was with an MOI of 50. (*a*)(i) Cells were challenged with G for 30 min then counted, or (ii) challenged with G for 30 min, washed to remove extracellular bacteria and left for a further 30 min before being counted: in either case, about 80% of cells contained G. (iii) as (ii) but cells were challenged again with G for 30 min after the washes: more cells were infected by G than in (i) and (ii). (iv) as (ii) but challenged with R for 30 min after the washes. The proportion of cells infected by G alone in (iv) was clearly less than in (i) or (ii), most of the difference being accounted for by cells infected by both G and R. (*b*) An equivalent set of experiments, but with G and R swapped. The infection rate of R is lower, but the same qualitative result holds. (*a*,*b*) (v) Gives the expected counts under the null hypothesis of independence of G and R infection (see main text). (*a*,*b*) Cells infected with both G and R, and uninfected cells were observed more frequently than expected (*p* < 10^−4^).

To determine whether reinfection occurs and thus influences the number of bacteria within the cell, we performed a set of experiments whereby BMDM were infected with G or *S.* Typhimurium SL1344 expressing ds-Red under the control of the P*ara*BAD arabinose-inducible promoter (referred to subsequently as R). In these sequential challenge experiments, 500 BMDMs were challenged with G for 30 min, then washed and challenged again with R for a further 30 min. Three different controls were performed: G was used for both challenges, no second challenge was performed or the experiment was terminated at 30 min (before the second challenge). Each challenge used an MOI of 50. Experiments were also performed where the order of infection with G and R was reversed, for completeness. After immunostaining for LPS O-antigen, infected cells were visualized and BMDM were categorized as containing only G, only R, both G and R, or as containing neither (i.e. uninfected). Each experiment was performed in triplicate, and pooled data are presented (figure 1*a,b*(i–iv)). R consistently infected fewer cells than G. Although we had seen no difference in growth curves for G and R, it could be that the toxicity of ds-Red is reducing fitness of R in the context of infection studies.

If reinfection were not possible, then dual infection of individual cells could happen only through a single event in which the BMDM was infected by both G and R simultaneously, which should be rare here because the initial population of bacteria are washed off before the second challenge. However, 418 of 1500 BMDM contained both G and R in the first series of experiments, and 431 of 1500 when the order of infection with G and R was swapped. The number of BMDM containing only G or R was correspondingly reduced in sequential challenge compared with any of the single challenge controls: this is consistent with BMDM undergoing sequential infections (see figure 1 for details).

**Figure 2.**
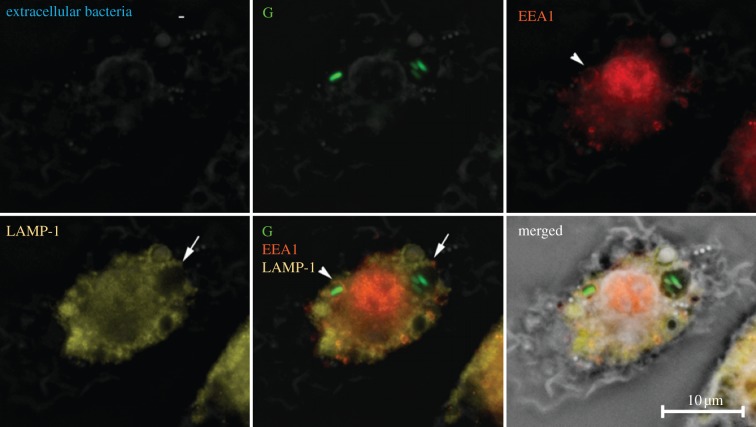
*Salmonella* Typhimurium are contained in *Salmonella*-containing vacuoles (SCVs) at different stages of maturation, indicating that reinfection events have occurred. Overnight cultures of *S.* Typhimurium SL1344 expressing GFP (G) were added to BMDM at an MOI of 100. Samples were fixed at 10 min post-infection and immunolocalization for extracellular bacteria was performed using anti-O5 antisera. The panels show the localization of extracellular G bacteria, the early phagosomal protein EEA1, the late phagosomal protein LAMP-1, the overlaid image of G, EEA1 and LAMP-1 and the merged picture of all the images in phase contrast. The arrows indicate SCVs of differing maturity containing *S.* Typhimurium. A total of 100 BMDM were assessed per experiment and this was repeated on three separate occasions.

We used microscopy to investigate the intracellular localization of the bacteria. Within the macrophage, *S.* Typhimurium resides in the SCV [12]. During the first steps of the maturation process, the early SCV expresses endosomal markers, such as endosomal antigen 1 (EEA1), but these early protein markers are rapidly replaced by late endosomal markers such as lysosomal glycoprotein 1 (LAMP-1) [12,13]. If multiple bacteria were to infect a cell in the same event, then they would reside in the same SCV. However, if two separate infection events happened at different times, then one might expect that the bacteria should reside within different and separate SCVs, expressing endosomal markers denoting different maturity within the same cell. To test this assumption, BMDMs were infected with G (at an MOI of 100) for 10 min and then immunostained for EEA1 and LAMP-1. In infected cells containing more than one *S.* Typhimurium, the bacteria were often contained within SCVs at different stages of maturation (figure 2), supporting the existence of a reinfection process.

**Figure 3.**
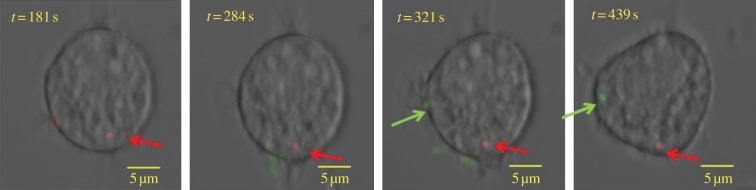
Direct evidence of reinfection using real-time confocal imaging. Overnight cultures of *Salmonella* Typhimurium SL1344 expressing either GFP (G) or ds-Red (R) were added to RAW264.7 macrophages. Cells were infected with R at an MOI of 50 for 15 min then, after three washes to remove any residual bacteria, an infected macrophage was identified and G was added at an MOI of 100. The infected cell was observed in multiple planes (Z-stacks) over time to confirm the presence or the absence of intracellular bacteria. In this figure, a range of confocal Z-stacks is merged together, and overlaid to the phase-contrast image obtained in transmission. The four images are from sequential timepoints: 181 s, 284 s, 321 s and 439 s. The first two images from 181 s and 284 s show the presence of the R bacterium within the macrophage. The third image at 321 s shows an extracellular G bacterium associating with the macrophage. In the final image at 439 s, the G bacterium is within the macrophage, along with the R bacterium, demonstrating a second infection event. Movies showing the Z-stacks separately are available as electronic supplementary material.

In order to gather direct evidence of reinfection events, we performed real-time confocal imaging of individual cells exposed to G and R bacteria. This technically challenging experiment required a change from BMDMs to the mouse-macrophage-like cell line RAW264.7, which is more susceptible to infection, thus increasing the chances of visualizing live infection events. G and R bacteria were added to 2 × 10^5^ RAW267.4 macrophages. Cells were challenged for 15 min with R at an MOI of 50, washed three times to remove any residual bacteria and then, after identifying and keeping in view an infected cell, G bacteria were added at an MOI of 100. The infected cells were observed in multiple planes (Z-stacks) over time to confirm the presence or absence of intracellular bacteria. Using time-lapse confocal microscopy, we unequivocally visualized the infection of a cell already infected with an R bacterium by a G bacterium. Still images of merged Z-stacks are shown (figure 3). Movies showing the Z-stacks separately are available as electronic supplementary material.

Having established experimentally that macrophages can be reinfected, a key question concerns whether infection alters macrophages in their susceptibility to further infection. The independence of separate infection events was tested using the sequential challenge data described earlier. Our null hypothesis was that infection by G and R would be independent; so a cell infected by G, say, would be as likely to be infected by R as a cell that was not infected by G. To find the expected distribution if the R and G infections were happening independently, we first note the percentage infected with G (ignoring the presence or the absence of R): in figure 1*a*(iv), 418 + 445 gives 57.5% for G. Similarly, for R, 418 + 246 gives 44.2%. Under a null hypothesis of independence of strains, we would expect 57.5% × 44.2% × 1500 = 382 BMDM infected with *both* strains. This expected value is calculated for each part of the distribution and given in figure 1*a*(v),*b*(v). In the observed data, there were 418 cells infected with both G and R. The probability of a difference at least this extreme happening by chance under independence of strains can be calculated using Fisher's exact test (one-tailed): *p* < 10^−4^ here (*p* < 10^−5^ for G and R swapped). This suggests that the strains are not behaving independently: there are disproportionately many dual infections, or equivalently, uninfected cells. There are four obvious mechanisms that would violate the null hypothesis: (i) new cells growing; (ii) cells being killed by infection; (iii) heterogeneity of cell susceptibility and (iv) infection changing the probability of further infection, the last two processes being central to this study.

### 2.2. Construction of a mathematical model to determine the contribution of the infection rate, intracellular bacterial growth and reinfection to the number of intracellular bacteria

**Figure 4.**
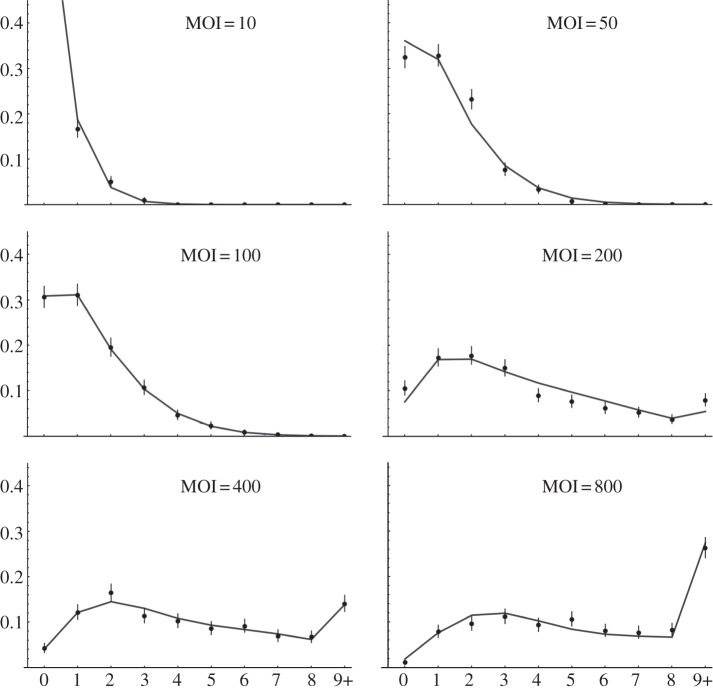
Bacterial count distributions at 10 min post challenge, different MOIs, with model fits. BMDM cells were infected for 10 min with G at a range of MOIs (10–800). The horizontal axis refers to the number of intracellular bacteria, with 9+ merged into one class, the vertical axis refers to the proportion of macrophages (from 1500 cells for each MOI), and the bars give the 95% CIs from multinomial models. The grey curves give the best-fitting model: two cell populations with differing susceptibilities, intracellular growth of bacteria, reduced rate of infection after first infection and no death of infected cells. Ten parameters in total were used (for all MOI together) and the fitted values are given in table 2.

Our experimental analysis challenged several of our original assumptions, leading us to construct a more detailed set of experiments and models to explore the dynamic interaction of *S.* Typhimurium with macrophages. We exposed BMDM to salmonellae at six different MOIs and counted the number of intracellular bacteria in each of 500 cells per experiment. A time point of 10 min p.i. challenge was chosen so that: (i) host cell replication would be negligible; (ii) extracellular bacterial growth would be negligible and (iii) bacterial killing of macrophages would not dominate the dynamics. The experiments were performed in triplicate, and the data were pooled, making a total of 9000 cells being counted (figure 4). At the lowest MOI, there were at most four bacteria per cell, with most of the cells remaining uninfected. At the highest MOI, many of the cells contained at least one bacterium by 10 min, and some cells contained many bacteria (20+).

These distributions contain a wealth of information on the different processes that shape intracellular bacterial counts. Models were developed in parallel with the experimental approach to dissect the different mechanisms shaping these distributions. The basic mathematical system is given by a Markov chain for the state of the cells, where 10 different states correspond to different numbers of intracellular bacteria: 0, 1, 2, 3, … , 8 or 9 or more. The truncation (more than nine bacteria per cell) is in keeping with the resolution of the data, and difficulty of accurately quantifying the number of bacteria in very heavily infected cells. The model for a single population of cells is given by

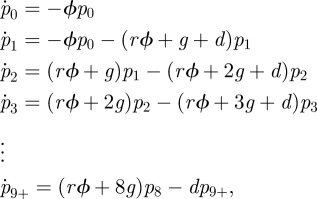

where *p_i_* denotes the probability that a cell contains *i* bacteria. The relative reinfection rate, *r*, is the multiplicative increase/decrease in infection rate for cells which are already infected—this can be set to 1 to make the reinfection rate the same as that for primary infection. The growth rate of intracellular bacteria is given by *g* and *d* is the death rate of infected cells. In the model, the dynamics of each cell is independent of the others: this is an approximation for the sake of tractability and parsimony, but we expect it to be reasonable for the short timescale modelled here. The raw infection rate **ϕ** is treated as constant within each experiment: implicitly this assumes that the extracellular bacterial numbers are relatively constant over the timescale of the experiment (i.e. during lag phase), however **ϕ** is treated as a function of MOI. The death rate means that these probabilities do not sum to unity (there is an implicit extra state: dead cells). The model was run for the length of time corresponding to the experiment, and then all values were re-normalized by the total—effectively, this is conditioning the probabilities on cell survival.

A simple modification was made to include the possibility of there being two distinct types of cell, one more susceptible than the other. Two additional parameters were needed: *a* is the proportion of the population that is more susceptible, and **ρ** is the factor by which they are more susceptible. The model was run twice, one exactly as above, and one with the **ρ** multiplying **ϕ**, and then they were pooled in proportion 1 − *a* and *a*, respectively, then finally the normalization was carried out.

A set of 16 candidate models were formed by including or excluding all combinations of four features (intracellular bacterial replication; death of infected cells; reinfection rate differing from first infection and one or two populations of cells). The features can be turned ‘on’ or ‘off’ by fixing parameters at zero (death and growth rates, or proportion of more susceptible cells) or one (relative reinfection rate). For each of the models, **ϕ** (the basic infection rate) was allowed to differ according to MOI, but all other parameters were assumed to be fixed across different MOIs.

Likelihoods were computed based on multinomial distributions: given the dataset (*c*_0_, *c*_1_, … ,*c*_9+_) of cell counts where *c_i_* is the number of cells containing *i* bacteria, then the probability of observing this dataset given the model and parameters is given by

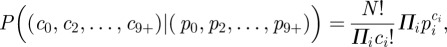

where *N* is the total of the *c_i_*. Taking logarithms of both sides (to simplify maximization as only the second term need be considered):

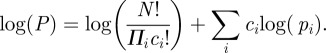



The model simulations and likelihood maximization were carried out in Mathematica v. 7 (Wolfram Research, Inc.).

Model selection was made using Akaike information criterion [14] (AIC; twice the number of fitted parameters minus twice the log-likelihood). A lower AIC corresponds to a better fit and/or a more parsimonious model. Table 1 gives *Δ*AIC: the AIC difference from the minimum AIC. *Δ*AIC of 0–2 indicates a model with substantial support, 4–7 of considerably less support and *Δ*AIC > 10 indicates essentially no support [14]. Akaike weights were also computed: these give proportions of weight, summing to one.

**Table 1.** Model selection. Blank entries (—) indicate the parameter was fixed at zero, and bracketed values indicate any other fixed values. The model with *Δ*AIC = 0 is selected.

	fitted	*Δ*AIC	weight	*g* (s^−1^)	*r*	*d* (s^−1^)	*a*	**ρ**
one population	*—*	1918.5	0	—	(1)	—	—	—
	*d*	499.1	0	—	(1)	1.1 × 10^−2^	—	—
	*r*	951.7	0	—	1.98	—	—	—
	*r, d*	93.0	0	—	0.41	3.7 × 10^−2^	—	—
	*g*	391.5	0	1.1 × 10^−3^	(1)	–	—	—
	*g, d*	393.5	0	1.1 × 10^−3^	(1)	8.9 × 10^−5^	—	—
	*g, r*	385.6	0	1.2 × 10^−3^	0.88	—	—	—
	*g, r, d*	95.0	0	<10^−8^	0.41	3.7 × 10^−2^	—	—
two populations	*—*	10.2	0	—	(1)	—	0.47	2.92
	*d*	12.2	0	—	(1)	2.0 × 10^−5^	0.47	2.91
	*r*	12.1	0	—	0.99	—	0.47	2.94
	*r, d*	11.3	0	—	0.83	1.2 × 10^−3^	0.51	2.87
	*g*	7.7	0.02	9.4 × 10^−5^	(1)	—	0.46	2.85
	*g, d*	9.7	0.01	9.4 × 10^−5^	(1)	<10^−8^	0.46	2.85
	*g, r*	0	0.71	2.5 × 10^−4^	0.84	—	0.48	3.00
	*g, r, d*	2.0	0.26	2.5 × 10^−4^	0.84	<10^−8^	0.48	3.00

Clear patterns emerge from this analysis: the only models with any support are exactly those including two populations of host cells and growth of bacteria. Within those models, there is strong support for a model with reinfection happening at a separate rate from initial infection (total weight: 0.97). At first glance, it appears that the inclusion of macrophage death is ambiguous. However, for the two relevant models that fit the death rate, the fitted rate *d* is indistinguishable from zero and the AIC differs only by the penalty for having an extra parameter. Hence, there is no support for including a macrophage death rate for this dataset. The best-fitting model output is shown together with the data in figure 4.

The model can be used to identify parameter values as well as testing different qualitative hypotheses. The likelihood was used to calculate credibility intervals or regions (from an uninformative prior). In practice, the large number of cells counted and classified (9000) means that the likelihood surface is very close to multivariate normal for a wide region around the maximum likelihood. The full set of fitted parameters for the selected model is given in table 2, together with their 99% credibility intervals (which are near symmetric).

**Table 2.** Fitted parameters. This table gives the fitted parameters for the best model (as described earlier). The fitted value is the maximum likelihood, and the ranges in brackets give the 99% credibility intervals.

parameter	fitted value and 99% CI
*a*	0.48 ± 0.05
*g* (s^−1^)	(2.51 ± 1.77) × 10^−4^
*r*	0.84 ± 0.13
**ρ**	3.00 ± 0.24
**ϕ**_10_ (s^−1^)	(2.34 ± 0.41) × 10^−4^
**ϕ**_50_ (s^−1^)	(1.01 ± 0.11) × 10^−3^
**ϕ**_100_ (s^−1^)	(1.19 ± 0.13) × 10^−3^
**ϕ**_200_ (s^−1^)	(3.23 ± 0.27) × 10^−3^
**ϕ**_400_ (s^−1^)	(4.22 ± 0.35) × 10^−3^
**ϕ**_800_ (s^−1^)	(5.51 ± 0.51) × 10^−3^

**Figure 5.**
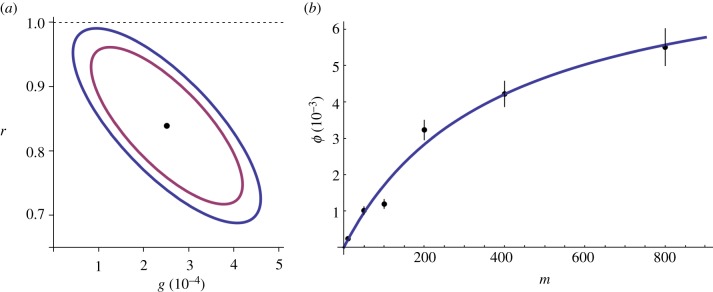
Key model parameters. Graphical plots of fitted parameter values: (*a*) gives the credibility region for *r* and *g* for 95% (inner ellipse) and 99% (outer ellipse). These two parameters are strongly interlinked, making it hard to estimate either parameter accurately on its own, but even the entire 99% region is contained within *r* < 1. (*b*) Gives the fitted infection rates for different MOI and the bars for their 99% CI. The relationship between infection rate and MOI may be linear for low MOI, but starts to flatten off for high MOI.

The relationships between the parameters can be explored using the covariance matrix as the likelihood function can be approximated well by a multivariate normal distribution close to the maximum-likelihood values. Unsurprisingly, there is a strong covariance between all the infection rates (changing any of the other parameters could potentially shift all of these in concert). The other strong relationship to emerge was the strong negative covariance between *r* (the relative reinfection rate) and *g* (intracellular growth). Their confidence ellipses are shown in figure 5*a*. It is intuitive that these parameters should be interlinked: both parameters are associated with mechanisms that shape the number of intracellular bacteria beyond the first infection: one through reinfection, and the other through growth of existing bacteria. The extremely wide range of MOIs used in the experiments will have helped us to distinguish the two mechanisms to some extent: in the high MOI regime, reinfection will be relatively more important, whereas in the low MOI regime, intracellular growth of bacteria may dominate. From the confidence regions, we can conclude that *r* is likely to be less than one; so it is possible to conclude that reinfection happens at a slower rate than first infection. Simply carrying out further replicates of similar experiments is unlikely to be effective in narrowing these estimates, as the difficulties of ambiguity between these two processes will remain. To identify the values individually to greater precision, ultimately, a novel experimental approach is required that allows some degree of distinction to be made between reinfection and growth.

The two-populations models were considered to explore whether the cells were homogeneous in terms of their susceptibility to infection, and it is a striking result that all the two-population models fit the data much better than all the one-population models. The fitted values indicate two populations of roughly equal size, with one three times more susceptible to infection than the other. It would be expected that a two-population model would fit better than a one-population model if there were actually two populations in the experiments, but this result could also follow if there were three or more populations. It is possible to fit increasingly complex models with more refinement in cell heterogeneity (and three populations does give a fractionally better fit, with proportions 41%, 43%, 16%, in the order of increasing susceptibility), but then in parallel, similar levels of detail should be explored for the other features that were established, i.e. considering the possibility that second infection alters susceptibility even further than first infection, or intracellular growth of bacteria is dependent on the density of intracellular bacteria. Analysing all of these possibilities in combination in a similar fashion to what is presented here would be technically very challenging, and would be in danger of exploring beyond the depth of detail that may be determined from our existing data.

A simple explanation for the model suggesting two populations of cells might be that the BMDMs contain a mixture of type 1 and type 2 macrophages that are differentially susceptible to infection with *S.* Typhimurium [15]. Primary macrophages generated in MCSF-containing media are predominantly type 1 [16]. Using flow cytometry analysis (for the type 2 macrophage marker CD206) [17], we confirmed that our primary BMDMs contained 80–90% type 1 and 10–20% type 2 macrophages. More type 2 BMDMs, as expected [16], are infected with *S.* Typhimurium (89.6% of cells) after challenge than the type 1s (39.6% cells), although whether this is due to increased bacterial proliferation or increased susceptibility to infection leading to increased numbers of reinfection events is unclear. The proportions of type 1 and 2 macrophages do not appear to correspond with the model fit of two populations of approximately equal size. Interestingly though, the proportions could be compatible with the three population model fit if the type 1 population itself was split into two subpopulations.

The infection rates **ϕ** increase with MOI, and exploring the precise relationship may yield insights into the infection process. A naive guess may be simply that the relationship is linear: infection is a simple mass-action process between bacteria and macrophages. Rather than a simple linear increase with MOI, however, the rate plateaus out for high MOI (figure 5*b*). There are several plausible reasons why the infection rate may start to saturate at high MOIs. Firstly, the cell is likely to have a maximum limit to its phagocytosis rate. An infection event may render the cell (or part of a cell) resistant to further infection for some period of time, for example, owing to changes induced in the membrane structure after phagocytosis or limitations in the availability of intracellular signalling molecules to mediate phagocytic events. Secondly, extreme MOIs may alter macrophage susceptibility to infection, probably owing to the effects of large amounts of bacterial products such as LPS or bacterial proteins that subvert intracellular signalling. A third possibility is that reinfection rates are more complicated than modelled here, and that this is having an artificial effect on overall infection rates. The model includes a single factor to describe the reduction in infection rates for any reinfection, but it could be that second and third infections could reduce the rate even further than the first. As there would be more multiple reinfection events at high MOI, this could manifest itself here as an apparent reduction in infection rate for increasing MOI.

We fitted a simple curve to our infection rates as a function of MOI, corresponding to the Hollings type II function response of predation in population ecology [18], or equivalently the Michaelis–Menten equation in enzyme kinetics, described by the function

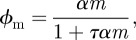

where *m* is the MOI, **α** corresponds to the infection rate per MOI in the low MOI limit (fitted here as 2.2 × 10^−5^ s^−1^) and **τ** can be interpreted as a processing time (fitted here as 121 s; figure 5*b*). Combining these, we find the MOI at which the processing time and searching time are roughly comparable is (**ατ**)^−1^ = 380.

For the two-population model, **α** must be modified by a factor (1 − *a* + *a*ρ**) to take into account the proportion of cells which have the extra susceptibility factor **ρ**. Finally, this gives an estimate of 4.2 × 10^−5^ s^−1^ for the infection rate per cell per MOI (for low MOI).

### 2.3. Construction of a physical model to determine encounter rates and probability of infection per encounter

An important factor is the rate of bacterial hits (average number of bacterial hits on one cell per unit of time), which is related to the MOI but is also dependent on the sample geometry, bacterial motility and cell density. To relate these parameters, a simple simulation of bacterial swimming was performed. The culture dish and culture medium depth were considered, as well as cell density and cross section, in order to determine the rate of bacterial hits onto the cell surface.

Bacteria are modelled as simple random Brownian walkers that make steps of equal length of 10 µm. After each step, a new random direction is chosen, and another step is made. This is a good approximation of the run and tumble behaviour seen in our live imaging study. If a bacterium hits either the walls (sidewall, dish bottom or surface of the culture medium) or a cell, then it is stopped and goes in a random direction. Each step in the simulation corresponds to very roughly 1 s (assuming a reasonable value of 10 µm s^−1^ for bacterial motility). The macrophages are modelled as half-spheres of radius *r* = 10 µm, randomly distributed on the bottom of the Petri dish. The dish (i.e. the available volume for cells) has radius *R* = 17.5 mm and the depth of culture medium was *h* = 2 mm.

**Figure 6.**
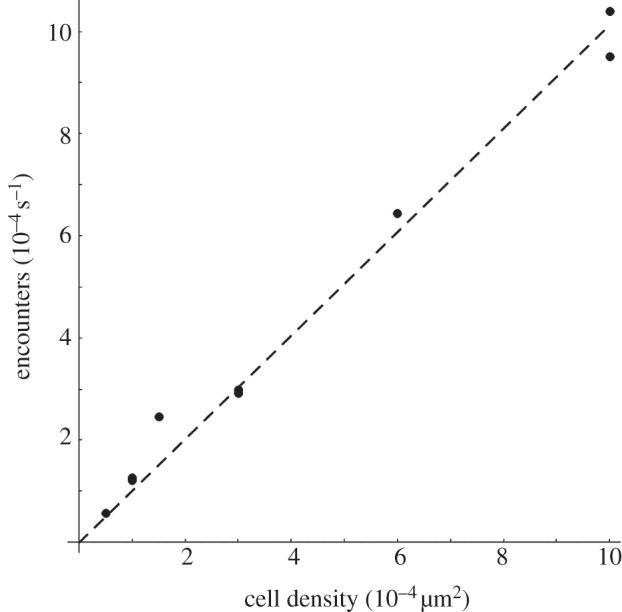
Relationship between cell density and encounter rate. The numerical model, including bacterial motility, sample geometry and cell density (parameters are described in the text), shows that in the experimental range of cell density the rate at which a bacterium encounters a cell depends linearly on the cell density. Markers indicate the results for different cell densities in the simulation. The dashed line is a linear fit, and the linear coefficient (1.01353) is used in the text to enable the comparison of experiments at different cell densities.

The simulation was run for a range of different cell densities (figure 6). The results are hit rates, i.e. the rate of cell hits per time-step for one bacterium. The range shown (up to 10^−4^ cells per µm^2^) corresponds to a small proportion of the culture dish base being covered by cells, and for this range the encounter rate appears to scale linearly with cell density with coefficient 1.01. For higher cell density, the linear relationship may not hold, but our experiments sit securely in the linear range.

**Figure 7.**
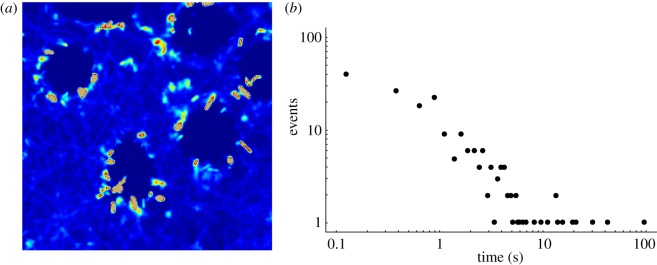
Bacteria can adhere to the cell membrane for a long time but rarely infect. Bacterial positions from the microscopy video were analysed both by recording tracks of individual bacteria, and by analysing the time a bacterium stays attached to a macrophage. (*a*) Shows the time-integrated signal from bacteria, with the colour of a pixel representing the accumulated signal (the overall probability that bacteria were at that position). There is a large probability of finding bacteria on the macrophage exterior, and inside the cells, there are, in general, no bacteria. Most of the bacteria adhering to the macrophages will swim away during this movie. In the intercellular medium, the bacteria are swimming in more or less straight lines, with random directions. There is no discernible chemotaxis. (*b*) Shows the frequency distribution of the time that a bacterium spends apparently sticking to a macrophage's surface. One hundred and ninety-two events were recorded, and 10 of these were over 10 s.

Bacterial positions were tracked in a microscopy video, in which the depth of focus was 10 µm. Figure 7*a* shows in false colours the localization of bacteria, integrated over time. The bacteria are highly motile, and while many tracks rapidly cross the field shown, some tracks spend a period nearly stationary, apparently sticking to the macrophage cell. However, very few of the bacteria that make contact with the cell end up infecting it. This is quantified by manually recording the attachment times, providing the data shown in the distribution plotted in figure 7*b*. One hundred and ninety-two events of bacteria observed sticking to cells were observed. The distribution in figure 7*b* is approximately exponential up to 10 s, beyond which there is a non-exponential tail of long attachment times. Most (95%) bacterium--macrophage encounters occur with an attachment time less than 10 s, in the exponential part of the distribution. The crossover to a different distribution of attachment times above 10 s implies a different bacterium–macrophage physical interaction, most likely a partial engulfment. Bacteria that remain attached to the cell for long periods of time (greater than 10 s) have a high probability of entering the cell. In the video analysed in figure 7*b*, only in 10 cases was the residential time more than 10 s, giving an upper bound of 5 per cent for the chance that a bacterium encountering a macrophage will infect it.

This rough consideration of the probability of infection per encounter can be combined with the encounter rate from the physical model (dependent on the cell density) to give an estimate for infection rate, which can then be compared for consistency with the fitted infection model (see earlier text). The experimental cell density was 5 × 10^−4^ per µm^2^, the linear fit gives an encounter rate of about 5 × 10^−4^ per bacterium per second. Combining this with a maximum probability of infection of 5 per cent (as estimated above), we find that the infection rate is 2.5 × 10^−5^ per second per bacterium. Although this differs from the corresponding estimate from the infection model (4.2 × 10^−5^, see earlier text), the comparison is still encouraging given the broad approximations made in the physical model, and the difficulties in parameter disambiguation in the infection model. The rates calculated by these two methods are of comparable magnitude, strongly supporting our estimates of infection rates and probability of infection.

## 3. Discussion

Here, we have used a tight iterative coupling of probabilistic models and a quantitative experimental approach to determine the probability of a macrophage becoming infected with *S.* Typhimurium after encountering the bacterium. It had previously been thought impossible to calculate either the probability of *S.* Typhimurium infecting a cell or the infection rate. Here, we have used iterations of *Salmonella* infection experiments with mathematical modelling versus analysis of real-time video microscopy of infection events to estimate these parameters. Despite the fact that *Salmonella* sp., are intracellular parasites of macrophages, our experimental observations interpreted via both the mathematical and physical models predict a low infection rate and a low probability of infection. The presence of negatively charged O-antigen in LPS of *S.* Typhimurium is anti-phagocytic and this has been suggested to cause electro-repulsion between cells and bacteria which would reduce bacterial–cell association and the likelihood of infection occurring [19]. Electro-repulsive effects are unlikely to occur in our experiments owing to the high concentration of sodium ions in tissue culture media, and here we see ready association of bacteria with macrophages, but infrequent infection events.

Our work also shows that an infected macrophage can be reinfected by a second *Salmonella* bacterium, although the rate of this reinfection is predicted to be lower than the rate for the first infection event. Our experimental analysis shows that relatively few macrophages in the population become infected and this, combined with our estimates of a low probability of infection, raises a number of important issues. Many studies, for example, assume that the macrophage responses measured after administering salmonellae to cells are caused by intracellular infection of most (or all) of the host cells in the culture. Our experimental data show that macrophages can encounter many bacteria without becoming infected, but that the bacteria do contact the cell, and therefore, host receptors on the cell surface, for example, Toll-like receptors, could easily be engaged and drive responses in the absence of intracellular infection of that particular cell.

Our combination of mathematical modelling with experimental analysis has revealed novel biological processes that occur when *S.* Typhimurium interacts with macrophages. A key question raised by our preliminary model was whether reinfection contributes to the total number of intracellular bacteria. Intracellular growth clearly contributes to the total number of intracellular bacteria, but recent work where intracellular growth was quantified saw no increase in intracellular bacterial proliferation until at least 3 h p.i. [8]. The concept of reinfection has not, so far, been considered in the context of contributing to the increase in number of bacteria seen within a macrophage. This could be because most experimental approaches consider time points of several hours p.i. and include a gentamicin step, at around 1 h p.i., after which the chances of reinfection occurring are likely to be low owing to extracellular bacterial death. At early time points during an *in vitro* infection, that is at less than 1 h p.i., gentamicin will not be present; so the possibility that reinfection could contribute to increases in intracellular bacterial number is potentially important. This may also be true during infections *in vivo*, where high MOIs can be achieved locally, for example, in an abscess. Our work shows unequivocally that reinfection occurs, and the combination of modelling and experimental data shows that reinfection contributes significantly to intracellular bacterial numbers in the early phase of an infection. Reinfection is, therefore, an important and previously overlooked mechanism and may contribute to total intracellular bacterial numbers in all *in vitro* infection studies.

The mathematical analysis identified that the observed data are best explained by a two-macrophage population model rather than having a homogenous population of cells. This suggests heterogeneity in cellular susceptibility to infection. Most biological studies analyse only the cells that have been infected by *S.* Typhimurium or consider studies where a population of cells have been pooled together and are all assumed to have been infected. Our work shows clearly that some cells are far more susceptible to infection than others. Heterogeneity or plasticity in macrophage phenotypes is a well-established concept with, at least, two types of macrophage characterized: classically activated (type 1) and alternatively activated (type 2) [15]. Type 1 macrophages are important for killing intracellular pathogens, whereas type 2 macrophages generate responses to parasites [15]. Our primary BMDMs contained over 80 per cent (type 1) and less than 20 per cent (type 2 macrophages). This provides a possible explanation for the heterogeneity suggested by the models, but quantitatively it is not consistent with the fit for proportions for the two-population model: the fit gave roughly equal-sized populations. Alternatively (and consistent with our model results), this disparity could be resolved if there were more than two populations, perhaps distinct populations within the type 1 macrophages, supporting the possibility of three or more subpopulations of cells, or indeed a spectrum of susceptibilities to infection with *S.* Typhimurium.

In conclusion, this study changes our assumptions about how *S.* Typhimurium infects macrophages. We have established that infection of a macrophage upon each individual contact with *S.* Typhimurium is a relatively rare event. If a cell becomes infected it may then be reinfected, but the initial infection makes the cell even less susceptible to further infection events. Different populations of macrophages that are differentially susceptible to infection are either present, or arise very quickly, during the infection process. A surprising corollary of our analysis is that the probability of a bacterium infecting a macrophage after they have encountered each other is low, even for bacteria that are in contact with a macrophage for several seconds.

## 4. Material and methods

### 4.1. Cell culture

Primary BMDMs were prepared as described [20]. RAW264.7 macrophages (from ECACC) were grown and maintained as described [21]. A preliminary analysis showed that RAW264.7 cells were more susceptible to infection than BMDMs, and RAW264.7 cells were therefore used for the live imaging analysis. Cells were plated onto six-well plates at a plating density of 4 × 10^5^ per well in a total of 2 ml of media (Sigma-Aldrich Ltd.).

### 4.2. Bacterial strains

*Salmonella* Typhimurium strain SL1344 expressing either green flourescent protein (JH3016) [22] or ds-Red fluorescent protein were used in this study. SL1344 was transformed with a pBAD18 plasmid containing ds-Red expressed from an arabinose promoter (a kind gift from D. W. Holden, Imperial College London, UK). Ds-Red expression was induced by adding 0.2 per cent l-(+)-arabinose to the bacterial broth. The growth curves in broth culture for the two strains of SL1344 were the same.

### 4.3. Challenge studies

Bacteria from frozen glycerol stocks were streaked onto fresh LB agar plates and incubated at 37°C overnight. A single bacterial colony was inoculated into LB broth and incubated in a shaking incubator overnight at 37°C. A 1 : 10 dilution of the overnight culture was incubated with shaking for 2 h at 37°C. Bacteria were centrifuged at 4300 *g* for 10 min and resuspended into an equal volume of phosphate-buffered saline (PBS). Optical density at 595 nm (O.D.595) was measured to determine the bacterial count, and the inoculum was diluted in PBS (200 µl final volume) to achieve the correct MOI. The bacterial viability and the MOI were confirmed by plating of culture dilutions. Experiments were conducted in culture medium without antibiotics to determine the bacterial-cell dynamics that occur during the infection phase of a traditional gentamicin protection assay. Macrophage death is characterized by the cell becoming detached from the plate or microscope slide. In microscopic analysis where cells are fixed, dead cells are removed by the washing steps described subsequently and, in live confocal analysis, dead cells were not analysed.

### 4.4. Immunostaining and microscopic analysis

In the microscopy and immunolocalization studies, cells were grown on coverslips. After challenge, the culture medium was replaced by 4 per cent paraformaldehyde in PBS for 10 min. Cells were washed twice with PBS for 15 min. In immunolocalization studies, cells were permeabilized with saponin (if required), incubated with 10 per cent normal goat serum for 10 min to block non-specific binding sites and then primary antibody was applied for 1.5 h at 4°C. Cells were washed twice in PBS for 15 min. Cells were incubated with secondary antibody conjugated to a fluorochrome for 30 min at room temperature, followed by two consecutive 15 min washes in PBS. Coverslips were inverted and mounted over glass slides with Vectashield and sealed. DAPI was used to visualize host cell nuclei. Control experiments were performed with rabbit antisera to *Salmonella* Vi antigen (for anti-*Salmonella* O5 LPS antisera) and rabbit or rat IgG for EAA and LAMP-1, respectively. Cells were imaged using a Leica DM600B fluorescence microscope with Leica FW4000 and AF6000 software. For live cell observation (figures 3 and 7), a Leica SP5 confocal microscope was used, with fast resonance scanning giving video rate imaging simultaneously in two fluorescence channels and the transmitted phase-contrast intensity.

The primary antibodies used were as follows: anti-*S.* Typhimurium O5-LPS (used at 1 : 200; Remel Ltd., KS, USA), anti-mouse CD11b (used at 1 : 200; Sigma-Aldrich, Gillingham, UK), anti-mouse EEA1 (used at 1 : 500; Abcam, Cambridge, UK), anti-mouse LAMP-1 (used at 1 : 200; Santa Cruz, Heidelberg, Germany), rabbit polyclonal IgG (used at 1 : 200; Abcam) and non-conjugated affinity-purified rat immunoglobulin IgG2a (used at 1 : 100; Abcam). The secondary antibodies (IgGs) used in this study were Alexa Fluor 350 conjugated goat IgG anti-rabbit IgG, Alexa Fluor 430 conjugated goat IgG anti-rabbit IgG, Alexa Fluor 568 conjugated goat IgG anti-rabbit IgG and Alexa Fluor 680 conjugated IgG goat anti-rat IgG. All secondary antibodies were purchased from Invitrogen, Paisley, Ltd (UK).
